# Human airway construct model is suitable for studying transcriptome changes associated with indoor air particulate matter toxicity

**DOI:** 10.1111/ina.12637

**Published:** 2020-01-23

**Authors:** Maria‐Elisa Nordberg, Martin Täubel, Pasi I. Jalava, Kelly BéruBé, Arja Tervahauta, Anne Hyvärinen, Kati Huttunen

**Affiliations:** ^1^ Department of Environmental and Biological Sciences University of Eastern Finland (UEF) Kuopio Finland; ^2^ Environmental Health Unit National Institute for Health and Welfare Kuopio Finland; ^3^ Cardiff School of Biosciences Cardiff Institute Tissue Engineering and Repair (CITER) Cardiff University Wales UK

**Keywords:** air‐liquid interface, immunotoxicity, in vitro, indoor air, normal human bronchial epithelial (NHBE) cells, particulate matter

## Abstract

In vitro models mimicking the human respiratory system are essential when investigating the toxicological effects of inhaled indoor air particulate matter (PM). We present a pulmonary cell culture model for studying indoor air PM toxicity. We exposed normal human bronchial epithelial cells, grown on semi‐permeable cell culture membranes, to four doses of indoor air PM in the air‐liquid interface. We analyzed the chemokine interleukin‐8 concentration from the cell culture medium, protein concentration from the apical wash, measured tissue electrical resistance, and imaged airway constructs using light and transmission electron microscopy. We sequenced RNA using a targeted RNA toxicology panel for 386 genes associated with toxicological responses. PM was collected from a non‐complaint residential environment over 1 week. Sample collection was concomitant with monitoring size‐segregated PM counts and determination of microbial levels and diversity. PM exposure was not acutely toxic for the cells, and we observed up‐regulation of 34 genes and down‐regulation of 17 genes when compared to blank sampler control exposure. The five most up‐regulated genes were related to immunotoxicity. Despite indications of incomplete cell differentiation, this model enabled the comparison of a toxicological transcriptome associated with indoor air PM exposure.

**Practical Implications**

- Human airway constructs cultured from primary bronchial epithelial cells are suitable for studying the toxicological effects of exposure to indoor air particulate matter.
- The tested experimental design provided enough high‐quality RNA for transcriptome analysis and showed activation of particularly immunotoxicological processes by indoor air PM.
- This airway epithelial model is a promising tool for comparing different indoor environments in environmental health research, also because it is well linkable to corresponding microbiome characterization of the exposure.

## 1 INTRODUCTION

Indoor environments harbor multiple different airborne exposure agents capable of entering the human respiratory system. Such exposure agents include gaseous pollutants (eg, volatile organic compounds (VOCs), nitrate and sulfur compounds, carbon monoxide, carbon dioxide, ozone, and radon), bioaerosols (eg, animal dust, pollen, dust‐mites, and microbes and their metabolites), and anthropogenic particles and fibers (eg, vehicle exhaust, combustion emissions, house dust, and tobacco smoke). Most of these types of inhalation exposure agents have been linked to indoor air problems in buildings. Exposure indoors always constitute a combination of agents, and it is likely that in many cases, synergistic effects of heterogeneous agents instead of a single component are contributing to the observed adverse health effects.^1^ For this reason, measuring the combined effects of multiple exposure agents rather than individual components is desirable in indoor environmental assessments.

Animal testing has been historically used for studying toxicity caused by microbial growth,^2^ coal combustion derived fine particulate matter,^3^ and dust chemicals^4^ present in the indoor environment. However, the respiratory system of rodents is anatomically and physiologically different from humans.^5^ Consequently, results from animal experiments are not directly comparable with the health effects observed in humans. Additionally, 3Rs (ie, replacement, refinement, and reduction) legislation and ethical considerations have restricted the use of animals in toxicological studies, resulting in replacement of in vivo methods with more affordable and easily implementable in vitro approaches.^5^ The epithelial layer of the respiratory system is the first barrier with which inhaled particles interact within the human body. Therefore, new cell culture models in inhalation toxicology aim to mimic the human respiratory epithelium.^6^, ^7^, ^8^

Human cell culture models are tissue‐engineered from either primary cells (ie, represent the tissue of origin) or secondary cell lines (ie, carcinoma‐derived or virus‐transformed). Co‐cultures of different secondary cell lines have also been utilized for several years in particulate matter (PM) exposure studies.^6^, ^9^ Immortalized secondary cell cultures derived from the bronchus and alveolar tissues are relatively common in inhalation toxicology. For instance, secondary human lung epithelial cells have been used for indoor PM_10_ toxicity studies^10^ and studying pro‐inflammatory responses of spores and hyphae.^11^ In secondary cell culture models, different types of cells are co‐cultured in either submerged or air‐liquid interface (ALI) configurations. However, the nature of secondary cell‐based systems has changed from their original state due to their carcinoid origin or immortalization process.^12^ Moreover, secondary cell line cultures are quite simple compared with genuine three‐dimensional human lung tissues, which consists of more than 40 distinct cell types^13^ with different functions. Consequently, comparing the study results from cell lines to those obtained from healthy human lung cells is not straightforward.^12^

Scientists have highlighted that the use of more complex cell culture or tissue models would enable us to study cell signaling and interaction in a more realistic way.^7^, ^8^ In addition to rather complex and costly developments such as different micro‐tissue chips, for example lung‐on‐a‐chip,^14^ stem cell‐derived models,^15^ and perfused organs,^16^, ^17^ companies have begun marketing primary cells extracted from the human nasal cavity, bronchi, and alveoli after surgical procedures or post‐mortem. These respiratory primary cells can be further differentiated to human airway tissue consisting of multiple cell types. For example, in vitro normal human bronchial epithelial (NHBE) cell model has been employed for toxicological testing^18^ and for inhalation modeling studies.^19^ However, human airway construct models can be used only once in a single exposure study due to their restricted division cycles. These cultures are also relatively expensive and laborious to maintain and differentiate.

The human bronchial epithelium is characterized as being a mucociliary phenotype featuring basal, goblet, Clara, ciliated, and intermediate cells.^18^, ^20^, ^21^, ^22^ Goblet or bronchiolar exocrine cells and secretory ducts protect the respiratory epithelium by forming and secreting lung surfactant. Moreover, lung surfactant protects cells via mucociliary clearance against inhalable particles and pathogens (eg, fungi, bacteria, and viruses). In toxicological studies, lung surfactant has been substituted by using water or physiological salt solutions containing phospholipids^23^, ^24^, ^25^ and surfactants.^26^, ^27^ In addition, some commercially available products, such as Survanta^®^ and Curosurf^®^, have been used.^28^, ^29^ Synthetic lung lining fluid (LLF) typically consists of water, phospholipids, fatty acids, sterols, proteins, and antioxidants that have been identified in human‐derived lung surfactant.^30^, ^31^

The aim of our study was to optimize a human airway construct model for studying indoor air PM toxicity using synthetic LLF to mimic the real human airway exposure more realistically. A commercially available toxicology transcriptome kit for 386 genes associated with toxicological responses was utilized for RNA sequencing. These measurements were paired with conventional toxicological and microscopical assessments, as well as with size‐resolved monitoring of airborne PM and microbial determinations.

## 2 MATERIALS AND METHODS

### 2.1 Sampling and monitoring indoor air particulate matter

Indoor air particulate matter (PM) samples were collected from a non‐complaint residential apartment located in an urban area in Eastern Finland by using NIOSH BC251 two‐stage bioaerosol cyclone sampler,^32^ stage 1, containing particles with cut‐off ≥2.1 µm in a 15 mL screw cap vial (National Institute for Occupational Safety and Health, NIOSH). An integrated sample was collected over the period of 7 days, intermittently for 12 hours each day, with total sampling time of 84 hours. The NIOSH sampler was operated at 10 L/min. Samples were stored at −20ºC before use. Airborne PM for microbial determination was collected using Button Inhalable Aerosol Sampler^33^ (SKC Inc.) onto polytetrafluoroethylene (PTFE) filter membranes (pore size 0.45 µm) (Merck Millipore) at 4 L/min daily during the same period. Filter samples were stored also at −20ºC until processing. Size‐resolved (PM_2.5_ to PM_10_) particle count and estimated mass concentrations (using a device‐specific assumption of particle mass) were monitored with Lighthouse Optical PM3016‐IAQ particle counter (Lighthouse Worldwide Solutions) during sampling. A proxy of the total particle mass in the samples was based on the device‐derived particle mass concentrations in the room air during sampling and the sampled air volume.

### 2.2 Sample preparation

#### 2.2.1 Indoor PM samples

PM samples collected with NIOSH sampler were suspended in cell culture medium (DMEM, 10% FBS, 1% Pen‐Strep, 1% L‐Glut) and mixed thoroughly for 30 seconds, sonicated for 15 minutes, and mixed again thoroughly for 30 seconds. Airway constructs were exposed to four doses (1:4, 1:8, 1:16, and 1:32) of indoor PM diluted in synthetic lung lining fluid (LLF). The estimated total particle mass in each of the doses is presented in the results.

#### 2.2.2 Synthetic lung lining fluid (LLF)

Composition of synthetic LLF is presented in Table 1. All reagents used in synthetic LLF were purchased from Sigma‐Aldrich.

**Table 1:** Composition of synthetic LLF. Reagents dissolved in acetonitrile indicated with light gray background and water‐soluble reagents indicated with dark gray background

Reagent	Concentration in LLF
1,2‐Dipalmitoyl‐sn‐Glycero‐3‐Phosphocholine	8 mg/mL
Phosphatidylglycerol	1 mg/mL
Phosphatidylethanolamine	0.2 mg/mL
Palmitic Acid	75 µg/mL
Glyceryl Tripalmitate	75 µg/mL
Cholesterol	0.2 mg/mL
α‐Tocopherol	1 µg/mL
Albumin Human	0.5 mg/mL
L‐Glutathione Reduced	0.5 mg/mL
L‐Ascorbic Acid	50 µg/mL
Uric Acid	25 µg/mL

Abbreviation: HBSS, Hank's balanced salt solution.

The recipe for synthetic LLF was adjusted based on previous studies.^30^, ^34^, ^35^, ^36^ First, non‐water‐soluble reagents were dissolved in acetonitrile (Sigma‐Aldrich), albumin, and uric acid in HBSS, and rest of the water‐soluble reagents in mQ‐water. Reagents were combined at room temperature and then mixed continuously (+37°C) for approximately five hours. After mixing, acetonitrile was evaporated under gaseous nitrogen flow in room temperature and pH adjusted to 7.25. Synthetic LLF was aliquoted and stored in −70°C before use.

### 2.3 Culturing human airway constructs

Human airway constructs were cultured and differentiated from normal human bronchial epithelial (NHBE) cells (Lonza^©^). The cells were isolated from non‐smoking Caucasian 62‐year‐old male. NHBE cells were defrosted and seeded at >3500 cells/cm^2^ into two T75 (75 cm^2^) cell culture flasks. Cells were incubated at 37ºC, 5% CO_2_ for 4 days (until 70%‐80% confluency). Transparent ThinCert^™^ cell culture inserts (0.336 cm^2^, pore size 0.4 µm) were treated in 24‐well plates (Greiner Bio‐One^®^) with 30 µg/mL rat tail collagen (Corning,) for 45 minutes before cell seeding. Cells were detached with 0.05% trypsin/EDTA solution (Lonza) and then neutralized using trypsin neutralizing solution (TNS) (Lonza). Cells were seeded 50 000 cells/insert and cultured for 3 days. B‐ALI^™^ Basal Media (Lonza) containing BEGM^™^ Bronchial Epithelial Cell Growth Medium SingleQuots^™^ Supplements and Growth Factors (Lonza) were changed daily. Cell cultures were transferred to air‐liquid interface (ALI) in B‐ALI^™^ Differentiation Media (Lonza) containing BEGM^™^ Bronchial Epithelial Cell Growth Medium SingleQuots™ Supplements and Growth Factors (Lonza) with additional 15 µg/mL retinoic acid (Sigma‐Aldrich), after which the cells were cultured in ALI for 22 days prior to exposure. Differentiation medium was renewed every other day. Transepithelial electrical resistance (TEER) of the tissue barrier was measured using EndOhm chamber and EVOM2^™^ resistance meter (World Precision Instruments), and cell cultures were evaluated using light microscope to assess differentiation and wellbeing of the cells.

### 2.4 Exposing human airway constructs to indoor PM

TEER was measured before exposure experiments and cell cultures with inadequate resistance (≤336 Ohm × cm^2^) were discarded. Airway constructs were exposed to four doses of indoor PM (1:4, 1:8, 1:16, and 1:32) in air‐liquid interface (+37°C, 5% CO_2_) for 24 hours. The PM samples were warmed up to +37°C and mixed thoroughly for 30 seconds just before applying 50 µL of each sample to the cells. 0.1 µg/mL Lipopolysaccharide from *Escherichia coli* O111:B4 (LPS) (Sigma‐Aldrich) in LLF was used as a positive control.

### 2.5 Analyses and RNA extraction

Transepithelial electrical resistance (TEER) was measured after the exposure to assess the integrity of the cell barrier. Chemokine interleukin‐8 (IL‐8) was analyzed from cell culture medium by using commercially available Human IL‐8 DuoSet cytokine kit (R&D Systems) and Victor^3^ plate reader (PerkinElmer) at 570 nm. Furthermore, protein concentration was analyzed from the apical wash using Bradford Reagent (Sigma‐Aldrich), 2 mg/mL Protein Standard (Sigma‐Aldrich), and Victor^3^ plate reader at 570 nm.

Seven human airway constructs were fixed overnight (2.5% glutaraldehyde, 0.1 mol/L phosphate buffer, pH 7.4) for further light and transmission electron microscopy (TEM) analysis. RNA was extracted from 50 human airway constructs using Qiagen RNeasy Plus Mini Kit (Qiagen) including DNase treatment. RNA samples were stored in −70 ºC and sent on dry ice to Sequencing Unit of Finnish Institute for Molecular Medicine (FIMM) for further RNA quality control and RNA sequencing.

### 2.6 Gene expression profiling by next‐generation sequencing (NGS)

#### 2.6.1 RNA quality assessment

Integrity and quantity of RNA was verified by Caliper GX RNA LabChip (Perkin Elmer) and Qubit RNA BR system (Thermo Fischer Scientific), respectively. Samples with 100 ng of high‐quality RNA (RNA score >8, DNase treated) were used for expression profiling by RNAseq.

#### 2.6.2 Library preparation for sequencing

QIAseq Targeted RNA panel Human Molecular Toxicology Transcriptome (Qiagen) of 386 genes associated with toxicological responses was used for first‐strand synthesis, molecular barcoding, gene‐specific amplification, sample indexing, and library preparation for targeted RNA sequencing according to instructions by manufacturer.

#### 2.6.3 Sequencing of RNA amplicons

Illumina HiSeq2000 platform was used for sequencing. All the samples were pooled in one lane of HiSeq flow cell with capacity of 238 million 100 bp reads.

### 2.7 Data analysis

The data were analyzed in GeneGlobe Data Analysis Center. In brief, after uploading the RNAseq data in FASTQ format the data were trimmed according manufacturer's instructions and aligned to GRCh38 reference genome using STAR RNA read mapper. After processing the alignments, molecular tags (unique molecular identifiers, UMIs) were counted for 386 genes including gDNA controls, reference genes, and a summary of data quality.

For further data analysis, the Secondary QIAseq Targeted RNA Panel Data Analysis Software (Qiagen) was used. After data normalization (trimmed mean of M, edgeR)^37^ and defining the groups for comparison, this software analyzed unique molecular index (UMI) counts to calculate changes in gene expression. Gene expression data were analyzed using Volcano plot analysis with minimum fold regulation of two (Student's *t* test, *P* < .05).

### 2.8 Quantitative and qualitative analyses of microbiota

PTFE filter samples collected with Button Inhalable Aerosol samplers were processed as previously described.^38^ In brief, after an initial mechanical lysis step on Mini‐Beadbeater‐16 (Biospec), DNA was extracted and purified using Chemagic DNA Plant kit (PerkinElmer, Baesweiler) on KingFisher DNA extraction robot (Thermo Scientific). Quantitative real‐time PCR (qPCR) was performed to assess bacterial and fungal levels in the samples, relying on previously established qPCR assays^39^, ^40^, ^41^, ^42^ and following the protocols described earlier.^38^ Bacterial PCR targeting the V4 region of the bacterial 16S rRNA gene^43^ (515F/806R) and amplicon sequencing were performed at a commercial sequencing partner LGC Genomics (Germany). A detailed description of the PCR protocol, amplicon sequencing, and sequence processing as performed with minor modifications in this current study is provided elsewhere.^44^ Rather than a nested PCR approach, direct PCR without prior pre‐amplification was performed for 30 cycles. Sequencing of a total of 22 samples (including bacterial and fungal mock communities, negative controls, and blank controls) was done on an Illumina MiSeq with V3 chemistry resulting in paired‐end reads with a length of 300 bp each. Processing of raw sequences into amplicon sequence variants (ASVs) was implemented in the standard DADA2 pipeline version 1.12.^45^ Taxonomy was assigned using SILVA^46^ database version 132. Alpha diversity measures, including observed ASVs and Chao1, Simpson and Shannon diversity indices, as well as taxa summaries were calculated in QIIME (Quantitative Insights Into Microbial Ecology)^47^ software version 1.9.1.

### 2.9 Microscopy

Sample preparations and transmission electron microscopy (TEM) imaging were undertaken at the SIB Labs (University of Eastern Finland, Kuopio, Finland).

#### 2.9.1 Transmission electron microscopy (TEM)

Samples were pre‐fixed with 2.5% glutaraldehyde in 0.1 mol/L phosphate buffer (pH 7.4) overnight. After pre‐fixing, samples were washed with 0.1 mol/L phosphate buffer (pH 7.4) for 20 minutes and post‐fixed with 1% osmium tetroxide in 0.1 mol/L phosphate buffer (pH 7.4) for 1 hour. Samples were washed again with 0.1 mol/L phosphate buffer (pH 7.4) for 20 minutes and then dehydrated gradually with ethanol and infiltrated overnight. Samples were polymerized to Laddin LX‐112 epoxy resin and cut into ultrathin sections (approximately 60‐70 nm), and post‐stained with 1% uranyl acetate for 30 minutes and lead citrate for 2 minutes. Finally, sections were imaged by using JEOL JEM‐2100F HR 200 kV field emission analytical electron microscope (JEOL Ltd), with an attached Olympus Quemesa camera and Olympus iTEM software (Olympus).

#### 2.9.2 Light microscopy (LM)

Semi‐thin resin sections of 1.0 µm were cut from TEM blocks and stained with toluidine blue.^48^, ^49^ Samples were imaged using Zeiss Axio Imager M2 light microscope (Zeiss), using the 40 × objective, Carl Zeiss AxioCam MRc high‐resolution color camera, and Axiovision Rel. 4.8 software (Zeiss).

### 2.10 Statistical analyses

Statistical analyses and figures were done using GraphPad Prism 8.1.1 (GraphPad Software). The normality of the data was tested with the Shapiro‐Wilk test,^50^ *P* < .05. For comparing TEER, chemokine IL‐8 secretion, and protein secretion of exposed airway constructs, controls (LLF, growth medium, and LPS exposed) were compared using ordinary one‐way ANOVA, Bonferroni's multiple comparisons test,^51^ *P* < .05 and PM exposed cells using two‐way ANOVA, Bonferroni's multiple comparisons test, *P* < .05.

## 3 RESULTS

### 3.1 Particulate matter concentrations and microbial content of PM samples

Particle concentrations in indoor air as well as microbial levels and composition were monitored during individual days of the sampling period. Based on the particle count and flow rate, we estimated the total particle mass in 7‐day integrate indoor PM sample used in the exposure. The estimated total particle mass concentration in the highest dose (dilution 1:4) used in the exposure experiment was 98 µg/mL, corresponding to a surface load of 15 µg/cm^2^. The mass concentrations of the further dilutions (1:8; 1:16; 1:32) were estimated to be 49 µg/mL (7.4 µg/cm^2^), 25 µg/mL (3.7 µg/cm^2^), and 12 µg/mL (1.9 µg/cm^2^), respectively. The airborne PM concentrations (PM_2.5_ and PM_10_) showed different diurnal cycles for weekend and working days, linked to activity patterns in the monitored apartment (Figure S1). PM concentrations, and bacterial and fungal levels as well as bacterial species richness varied considerably between sampling days (Table S1 and Figure S2). This supports the chosen approach to collect and analyze an integrated sample of 12‐hour sampling periods from seven subsequent days. The bacterial content of the 7‐day integrated sample was dominated by *Staphylococcus* and other genera that are likely of human origin.

### 3.2 Light microscopy and TEM imaging

**Figure 1:**
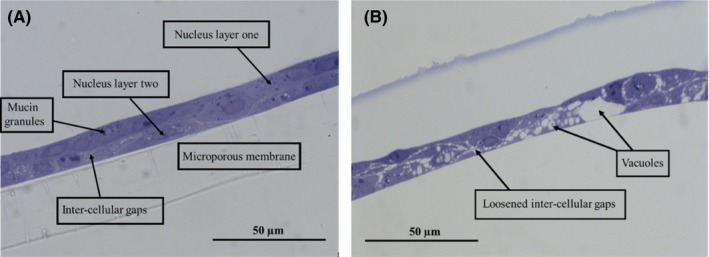
Toluidine blue‐stained semi‐thin sections observed by light microscopy (magnifications 400×) of (A) Control and (B) indoor air PM exposed (24 h, dose 1:4) human airway constructs

In light microscopy images, we located two nucleus layers, mucin granules, and inter‐cellular gaps (Figure 1A). In PM exposed cells, we observed loosened inter‐cellular gaps between cell layers and signs of cytolysis (ie, the dissolution or disruption of cells, especially by an external agent) throughout the cell layers following PM exposure (Figure 1B).

**Figure 2:**
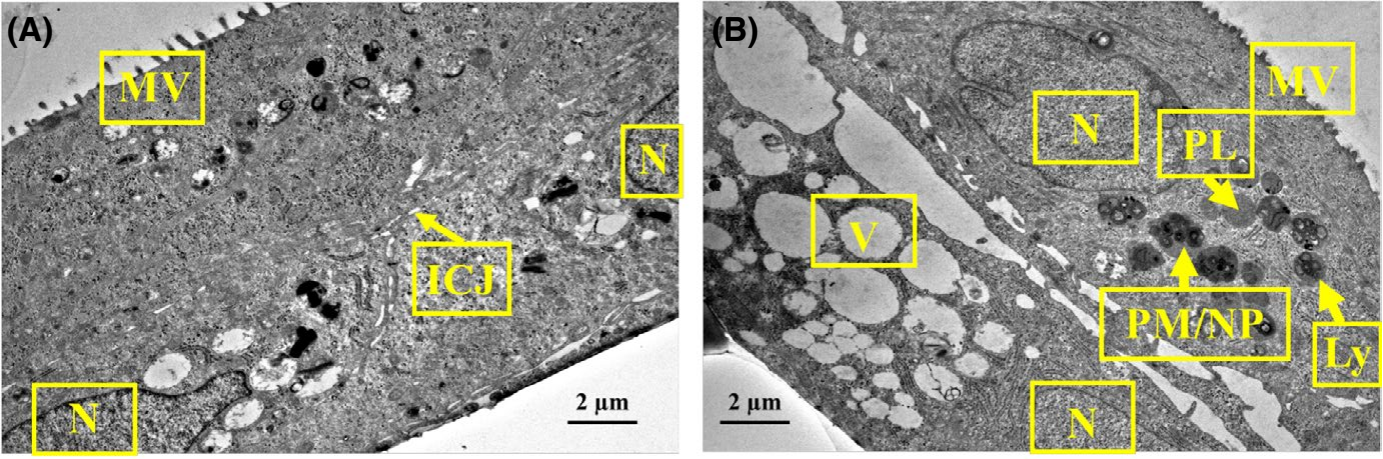
Transmission electron microscopy images (magnification 3000×) of human airway constructs exposed 24 h to (A) control and (B) indoor air PM (dose 1:4). Microscope JEM‐2100F, HV 200 kV and HFW 19.5 µm. Abbreviations: ICJ, inter‐cellular junction; Ly, lysosome; MV, microvilli; N, nucleus; PL, primary lysosome; PM/NP, particulate matter/nanoparticles; V, vacuole

In TEM images, the NHBE cell layer was determined to be 12 µm thick consisting of two nuclear layers (Figure 2A). Compared with control (Figure 2A), we observed multiple signs of cell distress after PM exposure (Figure 2B).

**Figure 3:**
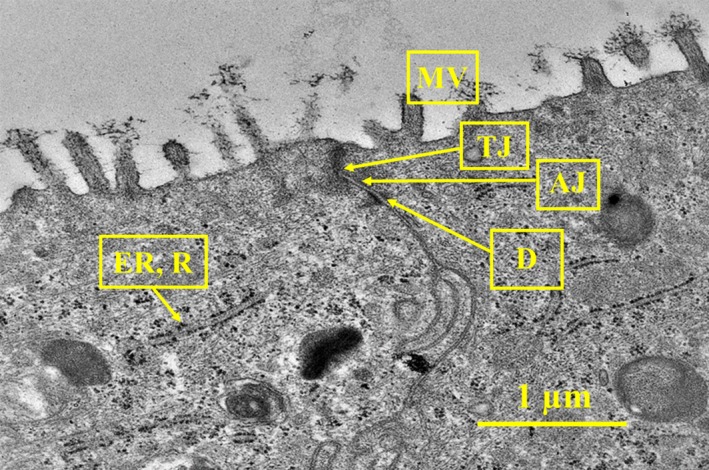
Transmission electron microscopy image (magnification 12 000×) of human airway construct control. Microscope JEM‐2100F, HV 200 kV, HFW 4.7 µm. Abbreviations: AJ, adherens junction; D, desmosome; ER, rough endoplasmic reticulum; MV, microvilli; N, nucleus; R, ribosomes; TJ, tight junction

Additionally, we observed microvilli, adherens junctions, and desmosomes in TEM images (Figure 3). No cilia, basal bodies anchoring cilia, glycocalyx around cilia/microvilli, pseudostratified morphology or maturation of columnar‐shaped ciliated and goblet cells were visible. We did observe 500 nm long microvilli without basal bodies and a 9 + 2 axoneme organization, which are characteristic of fully differentiated NHBE tissues (Figure 3).^18^

### 3.3 TEER measurements, protein secretion, and chemokine IL‐8 secretion

**Figure 4:**
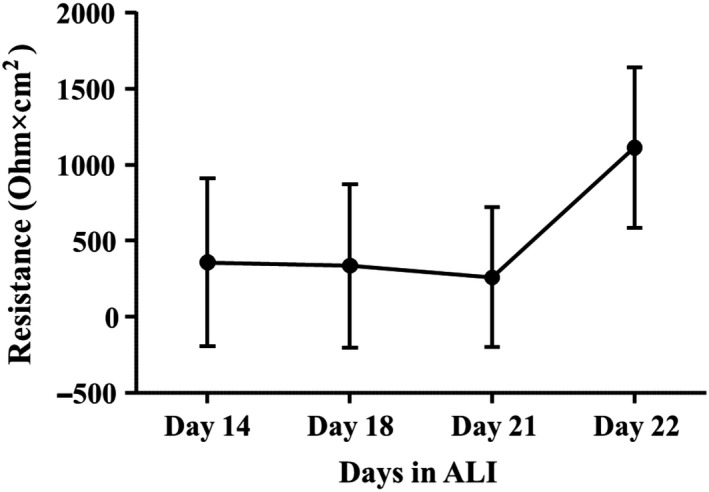
Mean resistance ± standard deviation (SD) of human airway construct barrier after 14 (n = 24), 18 (n = 24), 21 (n = 24), and 22 (n = 72) days in air‐liquid interface (ALI)

Human airway constructs were secreting surfactant after 10 days in air‐liquid interface (ALI), which is typical for lung epithelial tissue. Tissue resistance reached 360 Ohm × cm^2^ at Day 14 in ALI and increased up to 1100 Ohm × cm^2^ at Day 22 (Figure 4).

**Figure 5:**
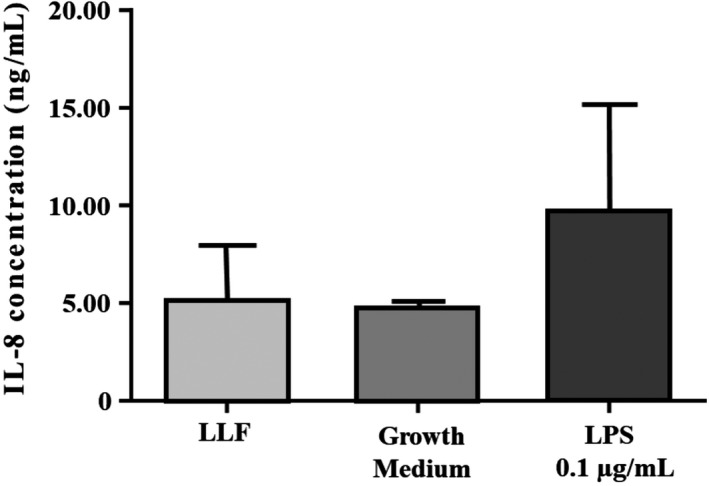
Chemokine IL‐8 concentration ± standard deviation (SD) after lung lining fluid (LLF) (n = 3), growth medium (n = 3), and LPS (n = 3) exposure (24 h)

IL‐8 secretion was stimulated after LPS exposure (Figure 5). However, chemokine IL‐8 production of LLF, growth medium, and LPS exposed cells did not differ from each other significantly (ordinary one‐way ANOVA, Bonferroni's multiple comparisons test, *P* < .05) (Figure 5).

**Figure 6:**
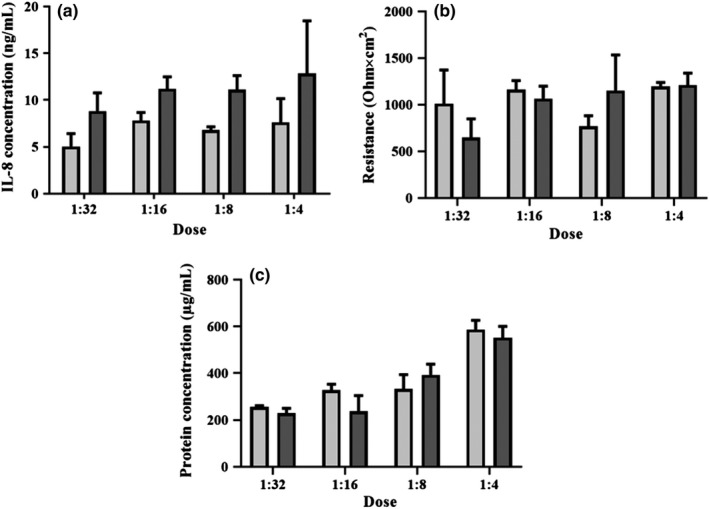
(A) Chemokine IL‐8 concentration ± standard deviation (SD), (B) Resistance ± SD of the human airway construct barrier, and (C) protein concentration ± SD in apical wash, after indoor PM exposure (n = 3) (24 h) compared with control (n = 3). Controls indicated with light gray and PM exposure with dark gray. Estimated total particle mass 15 µg/cm^2^ (1:4), 7.4 µg/cm^2^ (1:8), 3.7 µg/cm^2^ (1:16), and 1.9 µg/cm^2^ (1:32)

Chemokine IL‐8 secretion was consistently, but not statistically significantly increased due to PM exposure in different doses compared with control (Figure 6A). NHBE barrier resistance did not significantly decrease after indoor PM exposure (Figure 6B) and neither did the total protein concentration increase in apical wash after PM exposure (Figure 6C).

### 3.4 Gene expression profiling

**Figure 7:**
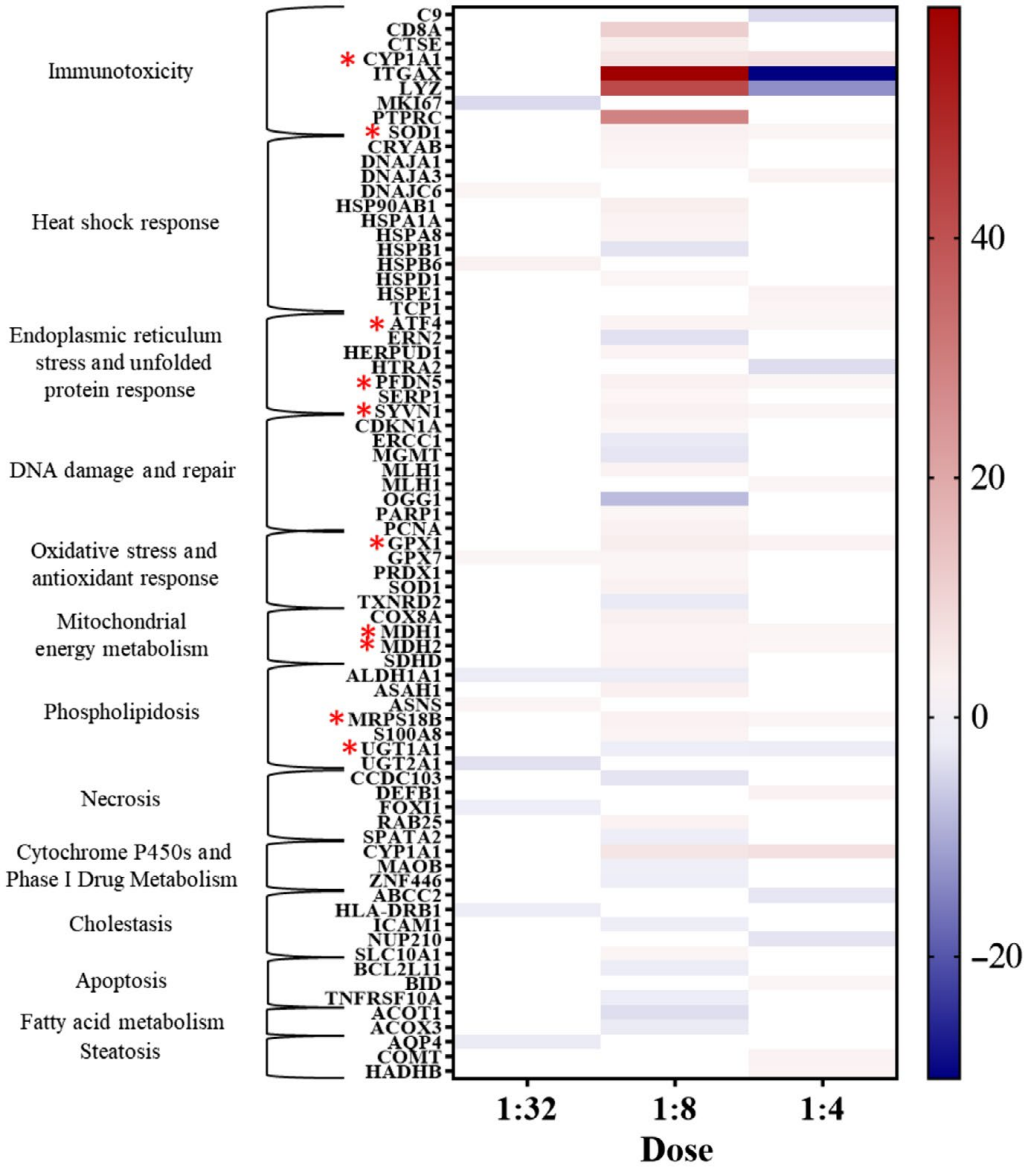
Heatmap of genes up‐ and down‐regulated (fold regulation, *P* < .05, fold difference ≥2) in human airway constructs after 24 h of indoor PM exposure. Red color indicates up‐regulation and blue color down‐regulation. Genes that were up‐ or down‐regulated in both 1:8 and 1:4 dosed airway constructs highlighted with red stars. Dose 1:16 was not included due to lack of statistically significant differences in transcription. Estimated total particle mass 15 µg/cm^2^ (1:4), 7.4 µg/cm^2^ (1:8), 3.7 µg/cm^2^ (1:16), and 1.9 µg/cm^2^ (1:32)

Multiple genes were up‐ and down‐regulated upon PM exposure of the cell cultures (Figure 7). There was no statistically significant change in transcription of cells when dosed 1:16. Ten genes that were significantly up‐regulated in cells dosed 1:4 were also up‐regulated with 1:8 dosing (Figure 7).

**Figure 8:**
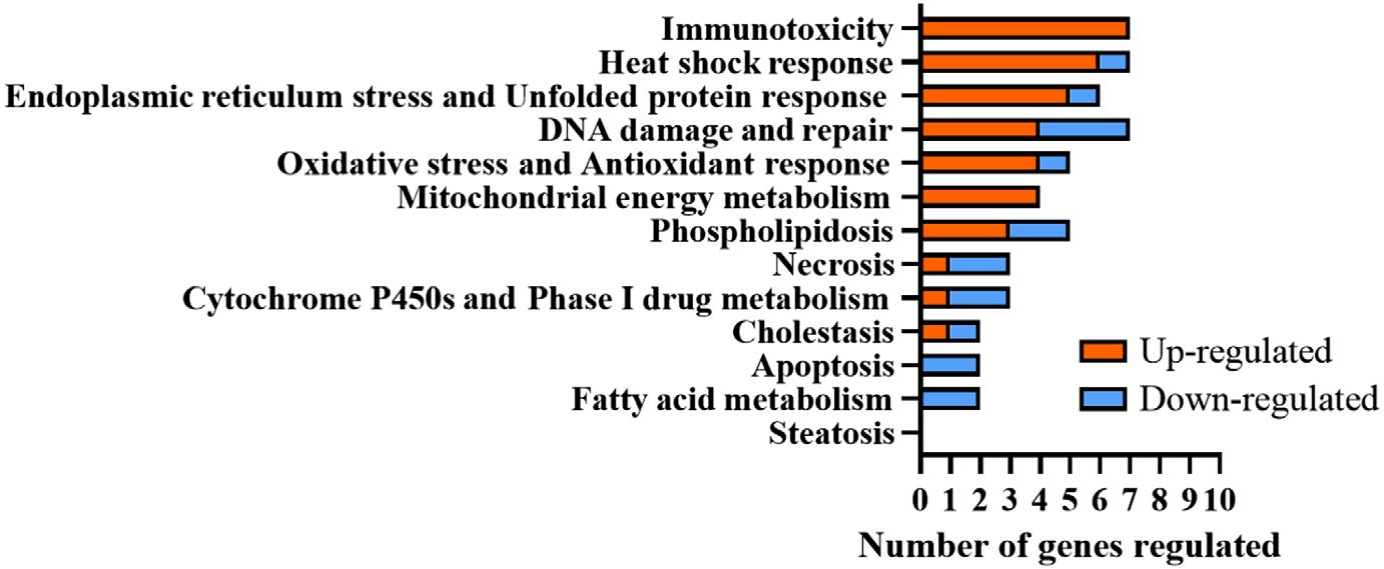
The number of up‐ and down‐regulated genes (fold regulation, *P* < .05, fold difference ≥ 2) in different cell function categories.^52^ Airway constructs exposed to 1:8 (estimated total particle mass 7.4 µg/cm^2^) indoor PM for 24 h

Two most up‐regulated genes, ITGAX and LYZ, in 1:8 dosed samples were strongly down‐regulated in 1:4 dosed samples. Transcription of cells dosed to 1:8 PM was most activated, up‐regulating the expression of 34 genes and down‐regulating 17 genes (fold regulation, *P* < .05, fold difference ≥2). Up‐regulation of 12 genes was doubled compared with control, and 22 genes were even more strongly up‐regulated. The number of up‐ and down‐regulated genes in different cell function categories^52^ in 1:8 dosed samples is presented in Figure 8.

Five genes (CD8A, CYP1A1, ITGAX, LYZ, and PTPRC) associated with immunotoxicity were among the most up‐regulated genes exposed to dose 1:8 indoor PM. Gene expression increased up to 60‐fold compared with control for the most impacted genes in the 1:8 PM exposure (Table 2). In addition, one oxidative stress‐related gene (OGG1) was up to eightfold down‐regulated compared with control (Table 2).

**Table 2:** Most regulated genes after 1:8 (estimated total particle mass 7.4 µg/cm^2^) PM exposure. Up‐ and down‐regulated genes with minimum of fivefold difference in gene expression compared with control were included

Gene	Expression compared with control (fold regulation)	Cell function category^52^
ITGAX	59	Immunotoxicity
LYZ	42	
PTPRC	29	
CD8A	11	
CYP1A1	6.0	
OGG1	−8.0	Oxidative stress and DNA damage and repair

Abbreviations: CD8A, T‐cell surface glycoprotein CD8 alpha chain; CYP1A1, cytochrome P450 1A1; ITGAX, integrin alpha‐X; LYZ, lysozyme C; OGG1, N‐glycosylase/DNA lyase; PTPRC, receptor‐type tyrosine‐protein phosphatase C.

## 4 DISCUSSION

When developing new methods for studying indoor PM toxicity, multiple factors such as representativeness of sampled PM and used in vitro or in vivo models need to be considered. In this study, we present a novel approach for assessing indoor PM toxicity, relying on measuring transcriptome changes in a panel of genes associated in toxicological processes in a human airway construct model. To the best of our knowledge, the human airway construct model has not been used for elucidating pulmonary toxicity caused by indoor air PM exposure before.

Our main aim was to determine whether a human airway construct model could be implemented to observe changes in transcriptome after indoor air PM exposure. For this purpose, PM doses adequate for inducing cell activation but not acutely toxic for the constructs are required. This was achieved as denoted by (a) activated secretion of chemokine IL‐8, (b) sub‐acute toxicity assessed as total protein in apical wash, and (c) loss of tissue integrity. The airway constructs provided sufficient quantity of high‐quality RNA after PM exposure, enabling us to sequence RNA by a commercially available targeted RNA toxicology panel kit. In contrast, the response to the *Escherichia coli O111:B4 (LPS)* used as a positive control, was relatively low, suggesting that cultured tissues are less sensitive than secondary cell cultures. This is probably due to the presence of protective elements such as mucus‐producing cells, microvillus, and multilayered cells. In future studies, we will aim to test whether addition of macrophages in the airway model would increase the sensitivity of the model.

After indoor PM exposure, we observed up‐ and down‐regulation of multiple genes such as ITGAX, LYZ, PTPRC, CD8A, CYP1A1, and OGG1. Strikingly, all five most up‐regulated genes were associated with immune response. ITGAX, the gene that was most up‐regulated, encodes a protein which mediates cell‐cell interactions during immune reaction and is associated with monocyte adhesion and chemotaxis.^53^ LYZ, another highly up‐regulated gene, encodes lysozyme present in tissue fluids and has mainly bacteriolytic function.^54^ Interestingly, both of the most up‐regulated genes (ITGAX and LYZ) in PM exposed cells at the second highest dose level (dose 1:8) were the most down‐regulated genes at the highest dose level (dose 1:4), possibly indicating a feedback mechanism quenching the response as the PM dose increases. The most up‐regulated genes included also tyrosine‐protein phosphatase coding PTPRC, which is essential in T‐cell activation^55^ and CD8A, which is associated with antigen processing and presentation,^56^ signaling pathways of cell surface receptors,^57^ differentiation of cytotoxic T cells,^57^ and immune response.^58^ The fifth of the most up‐regulated genes, CYP1A1, is associated with immunotoxicity^52^ and xenobiotic metabolism.^59^ It is also known to be up‐regulated after exposure to polycyclic aromatic hydrocarbons,^60^ which could be relevant in indoor settings due to exposure to indoor combustion particles (eg, from candles or fireplaces) or alternatively due to outdoor aerosol infiltration to indoor environment. In contrast to these five genes, OGG1 was clearly down‐regulated. OGG1 is associated with protection against oxidative stress,^61^ acute inflammation responses,^62^ and DNA repair.^63^ Since PM was sampled from a non–moisture‐damaged, complaint‐free apartment, we did not expect to see highly toxic effects on airway constructs. Nevertheless, PM exposure induced up‐ and down‐regulation of several genes associated with toxicological processes.

Synthetic lung lining fluid was not acutely toxic for airway constructs, making it a good candidate for carrier buffer to be used in future exposure studies. Arguably, the cells lose their direct contact with air during the exposure experiment in our model. However, by using synthetic lung lining fluid as a carrier buffer for the PM, we can mimic the real‐life situation where a film of similar fluid normally covers the cells of the airways and the secretion of the mucus is further increased during inflammation or irritation. We noted that LLF composition and subsequently its effects on airway constructs may vary due to challenges in standardizing the LLF preparation process. Consequently, we highly recommend testing toxicity of each batch before applying synthetic LLF on airway cells. We observed also indications that the composition and toxicity may vary due to multiple thawing cycles and extended storing even when frozen. Without further and detailed testing, maximum of 2 months of storage in −70°C and one thawing cycle is recommended based on our observations. Due to long storing and multiple thawing cycles, LLF tends to crystallize and form separate phases, therefore becoming potentially harmful for the cells.

Light microscopy images indicated that the airway constructs were not completely differentiated. When differentiated, pseudostratified airway epithelium should contain tight and adherens junctions, desmosomes, different cell types, such as Goblet, ciliated, Clara, intermediate and basal cells after 18‐30 days in ALI.^18^, ^19^ In pseudostratified airway epithelium, mature cilia (length approximately 6 µm and width 0.3 µm), longitudinal microtubules inside the cilia forming 9 + 2 axoneme arrangement, and microtubules emerging from basal bodies are hallmarks of the differentiation process.^64^, ^65^ In this study, we were able to identify tight junctions, adherens junctions, desmosomes, microvilli, mucin granules, and two cell layers but not fully differentiated structures nor glycocalyx among cilia and microvilli. Interestingly, also free and endoplasmic reticulum (ER) bound ribosomes were identified in TEM images, which may be due to active protein synthesis following exposure to indoor PM. Mucus secretion on Day 10 in ALI and mucin granules pictured in light microscopy images likely indicate the presence of immature Goblet cells.^18^, ^64^ Instead of forming a columnar epithelium with pseudostratified morphology, cells formed a stratified squamous barrier. The metaplastic squamous differentiation without columnar cell layer was corroborated by light and electron microscopy. In addition, thickness of the cultures (15‐20 µm) indicated incomplete differentiation, as fully pseudostratified NHBE cultures have been reported to be 40‐50 µm thick.^18^, ^19^ Multiple factors, such as collagenization,^18^ nutrient deficiency, retinoic acid deficiency,^66^ temperature, handling, cell count, viability, seeding efficiency, cell harvesting protocols, variability between donors,^19^ cryogenic storage of donor cells, and cell doubling time, may have affected the differentiation process.

In this study, we measured transepithelial resistance values of 1100 Ohm × cm^2^ in differentiated pulmonary epithelium. Typically, fully differentiated NHBE cultures have resistance of 2500‐3000 Ohm × cm^2^ 67 although also significantly lower resistance values of 600‐900 Ohm × cm^2^ have been reported.^19^ In commercially available, readily differentiated NHBE cell cultures TEER values of 500‐600 Ohm × cm^2^ have been reported.^68^ Importantly, multiple factors such as thickness of the tissue, existence of tight junctions, temperature, and electrode position affect TEER values. This implies that the assessment of the extent of the tissue differentiation cannot be based on TEER measurements only, as the reported values vary also between fully differentiated constructs.

Transcriptomes of fully differentiated NHBE constructs and human airway epithelium in vivo are found to be similar,^69^ but obtaining fully differentiated constructs remains challenging. When using partially differentiated constructs in exposure studies, some factors possibly affecting the response to xenobiotic exposure should be considered. For example, transcriptional profile^70^ and microRNA expression^71^ of airway constructs change during differentiation process, and fully differentiated airway constructs have been reported to have a greater metabolic potential than non‐differentiated NHBE cells.^72^ Despite these limitations, non‐differentiated NHBE cultures have been successfully used for toxicological research.^73^, ^74^ Although not fully differentiated, our human airway constructs demonstrated multiple characteristics typical for differentiation and tissue morphology close to normal human airway epithelium. Importantly, we showed that the exposed constructs react reproducibly to indoor PM, indicating that our model is useful in studying indoor air PM toxicity.

The particle count and microbial content of intermittently occupied indoor spaces is known to fluctuate.^75^, ^76^ Our findings confirm the daily variation in the levels of particles and microbes linked to activity patterns, which prompted us to use multiple day integrate PM samples for toxicity testing. The bacterial content of indoor air in the studied apartment was dominated by human associated taxa, in line with previous studies showing that human‐derived bacteria dominate the bacterial content in house dust.^77^

Aerosol samples can be collected in multiple ways when studying indoor PM including microbial and viral particles.^78^ Filter‐based sampling, which requires downstream processing that includes extraction of PM from the filters, may selectively modify the sample composition. This is why we believe that our approach to collect the samples with cyclone‐based, active method into sampling tubes, from which particulate matter can be readily resuspended into synthetic lung lining fluid, represents inhaled PM in a less modified and more realistic way. As a downside of this method, weighing the sampled material is not technically feasible, so we had to assess the dose‐response relationship by testing a series of dilutions of the collected particulate matter. However, we were able to estimate the total particle mass based on the airborne particle count and the flow rate during the sampling. Even though this provides only crude estimates of the mass concentrations, the levels (12‐98 µg/mL) corresponded the doses used in earlier studies where cultured alveolar epithelial cells were exposed to urban air inhalable PM (25‐300 µg/mL).^79^

Our study describes the characteristics of indoor PM from one apartment, which serves well our aim to test the usefulness of this experimental model in a real‐life situation. However, the choice to analyze a pre‐defined panel of genes of toxicological relevance instead of a complete transcriptome limited the scope of conclusions we were able to make based on the data. Nevertheless, we are confident that the selection of the genes in this panel represents different toxicological pathways well. Moreover, when working with a very limited number of samples, a targeted panel is likely a more sound option than collecting non‐targeted screening data that increase uncertainty in data interpretation. The approach presented here will enable us to study associations between toxicological responses and microbiota characteristics in different types of indoor environments in the future. Follow‐up of this work will aim at comparing pulmonary toxicity caused by indoor PM sampled from multiple non–moisture‐damaged and moisture‐damaged houses (REMEDIAL consortium).

## 5 CONCLUSION

Here, we demonstrated that human airway constructs are highly useful in studying indoor air PM toxicity, while our work acknowledges that obtaining fully differentiated airway constructs remains challenging. The studied indoor PM exposure levels, with PM collected from a non‐complaint residential home, were not acutely toxic for airway constructs, but induced up‐ and down‐regulation of several genes associated with toxicological responses. Most up‐regulated genes were related to different functions of immune defense. This exposure model enables the comparison of transcriptome related to indoor air PM exposures and can be matched with microbiota determinations to study interactions with the toxicological responses.

## CONFLICT OF INTEREST

The authors declare no conflict of interest.

## AUTHOR’S CONTRIBUTIONS

Nordberg, Täubel, and Huttunen were involved in the design of the study and contributed substantially to collecting and interpreting data and writing the manuscript. Jalava helped planning and synthesizing lung lining fluid for exposure studies. Tervahauta contributed to planning the acquisition of transcriptome data. BéruBé contributed to interpreting microscopy images. Hyvärinen contributed to conception and design of the study as the leader of the REMEDIAL consortium. All authors revised and approved the final manuscript.

## References

[1] Korkalainen M , Täubel M , Naarala J , et al. Synergistic proinflammatory interactions of microbial toxins and structural components characteristic to moisture‐damaged buildings. Indoor Air. 2017;27(1):13‐23. 26806918 10.1111/ina.12282

[2] Jussila J , Pelkonen J , Kosma VM , Mäki‐Paakkanen J , Komulainen H , Hirvonen MR . Systemic immunoresponses in mice after repeated exposure of lungs to spores of *Streptomyces californicus* . Clin Diagn Lab Immunol. 2003;10:30‐37. 12522036 10.1128/CDLI.10.1.30-37.2003 PMC145275

[3] Yu J , Tang Y , Xu J . Effects of indoor coal fine particulate matter on the expression levels of inflammatory factors in ovalbumin‐induced mice. Toxicol Res. 2019;8(1):57–66. 10.1039/c8tx00221e PMC6334494 30713661

[4] Carlsson G , Pohl J , Athanassiadis I , Norrgren L , Weiss J . Thyroid disruption properties of three indoor dust chemicals tested in silurana tropicalis tadpoles. J Appl Toxicol. 2019;39:1248‐1256. 31066086 10.1002/jat.3810

[5] Huttunen K , Korkalainen M . Microbial secondary metabolites and knowledge on inhalation effects In: Viegas C, Viegas S, Gomes A, Täubel M, Sabino R, eds. Exposure to Microbiological Agents in Indoor and Occupational Environments. Springer: Cham; 2017:215‐216.

[6] Alfaro‐Moreno E , Nawrot TS , Vanaudenaerde BM , et al. Co‐cultures of multiple cell types mimic pulmonary cell communication in response to urban PM_10_ . Eur Respir J. 2008;32(5):1184‐1194. 18653652 10.1183/09031936.00044008

[7] Klein SG , Hennen J , Serchi T , Blömeke B , Gutleb AC . Potential of coculture in vitro models to study inflammatory and sensitizing effects of particles on the lung. Toxicol In Vitro. 2011;25:1516‐1534. 21963807 10.1016/j.tiv.2011.09.006

[8] Müller L , Riediker M , Wick P , Mohr M , Gehr P , Rothen‐Rutishauser B . Oxidative stress and inflammation response after nanoparticle exposure: differences between human lung cell monocultures and an advanced three‐dimensional model of the human epithelial airways. J R Soc Interface. 2009;1:S27‐40. 10.1098/rsif.2009.0161.focus PMC2843981 19586954

[9] Klein SG , Serchi T , Hoffmann L , Blömeke B , Gutleb AC . An improved 3D tetraculture system mimicking the cellular organization at the alveolar barrier to study the potential toxic effects of particles on the lung. Part Fibre Toxicol. 2013;10:31. 23890538 10.1186/1743-8977-10-31 PMC3733942

[10] Marchetti S , Longhin E , Bengalli R , et al. In vitro lung toxicity of indoor PM10 from a stove fueled with different biomasses. Sci Total Environ. 2019;1(649):1422‐1433. 10.1016/j.scitotenv.2018.08.249 30308911

[11] Øya E , Afanou AKJ , Malla N , et al. Characterization and pro‐inflammatory responses of spore and hyphae samples from various mold species. Indoor Air. 2018;28(1):28‐39. 28922584 10.1111/ina.12426

[12] Carter M , Shieh J . Cell Culture Techniques. Guide to Research Techniques in Neuroscience, 2nd edn. ‎Cambridge: Academic Press; 2015.

[13] Franks TJ , Colby TV , Travis WD , et al. Resident cellular components of the human lung: current knowledge and goals for research on cell phenotyping and function. Proc Am Thorac Soc. 2008;5:763‐766. 18757314 10.1513/pats.200803-025HR

[14] Huh DD . A human breathing lung‐on‐a‐chip. Ann Am Thorac Soc. 2015;2015(1):S42‐S44. 10.1513/AnnalsATS.201410-442MG PMC5467107 25830834

[15] Huang SX , Islam MN , O'Neill J , et al. Efficient generation of lung and airway epithelial cells from human pluripotent stem cells. Nat Biotechnol. 2014;32(1):84‐91. 24291815 10.1038/nbt.2754 PMC4101921

[16] Nakata Y , Dahms TE . Triolein increases microvascular permeability in isolated perfused rabbit lungs: role of neutrophils. J Trauma. 2000;49:320‐326. 10963546 10.1097/00005373-200008000-00021

[17] Steinhorn RH , Gordon JB , Tod ML . Site‐specific effect of Guanosine 3’,5’‐cyclic monophosphate phosphodiesterase inhibition in isolated lamb lungs. Crit Care Med. 2000;28(2):490‐495. 10708189 10.1097/00003246-200002000-00034

[18] Prytherch Z . An in vitro NHBE Model of the Human Bronchial Epithelium for Toxicological Testing. Cardiff: Cardiff University; 2010.

[19] Rayner RE , Makena P , Prasad GL , Cormet‐Boyaka E . Optimization of normal human bronchial epithelial (NHBE) cell 3D cultures for in vitro lung model studies. Sci Rep. 2019;9:500. 30679531 10.1038/s41598-018-36735-z PMC6346027

[20] Bals R . Cell types of respiratory epithelium: morphology, molecular biology and clinical significance. Pneumologie. 1997;51(2):142‐149. 9157452

[21] Crystal RG , Randell SH , Engelhardt JF , Voynow J , Sunday ME . Airway epithelial cells: current concepts and challenges. Proc Am Thorac Soc. 2008;5(7):772–777. 18757316 10.1513/pats.200805-041HR PMC5820806

[22] Prytherch Z , Job C , Marshall H , Oreffo V , Foster M , BéruBé K . Tissue‐specific stem cell differentiation in an in vitro airway model. Macromol Biosci. 2011;11(11):1467‐1477. 21994115 10.1002/mabi.201100181

[23] Davies NM , Feddah MR . A novel method for assessing dissolution of aerosol inhaler products. Int J Pharm. 2003;255:175‐187. 12672613 10.1016/s0378-5173(03)00091-7

[24] May S , Jensen B , Weiler C , Wolkenhauer M , Schneider M , Lehr CM . Dissolution testing of powders for inhalation; influence of particle deposition and modeling of dissolution profiles. Pharm Res. 2014;31:3211‐3224. 24852894 10.1007/s11095-014-1413-4

[25] Son YJ , McConville JT . Development of a standardized dissolution test method for inhaled pharmaceutical formulations. Int J Pharm. 2009;382:15‐22. 19665533 10.1016/j.ijpharm.2009.07.034

[26] Buttini F , Miozzi M , Balducci AG , et al. Differences in physical chemistry and dissolution rate of solid particle aerosols from solution pressurized inhalers. Int J Pharm. 2014;465:42‐51. 24491530 10.1016/j.ijpharm.2014.01.033

[27] Rohrschneider M , Bhagwat S , Krampe R , Michler V , Breitkreutz J , Hochhaus G . Evaluation of the transwell system for characterization of dissolution behavior of inhalation drugs: effects of membrane and surfactant. Mol Pharm. 2015;12:2618‐2624. 26091361 10.1021/acs.molpharmaceut.5b00221

[28] Pham S , Wiedmann TS . Dissolution of aerosol particles of budesonide in Survanta, a model lung surfactant. J Pharm Sci. 2001;90:98‐104. 11064383 10.1002/1520-6017(200101)90:1<98::aid-jps11>3.0.co;2-5

[29] Wang L , Castranova V , Mishra A , et al. Dispersion of single‐walled carbon nanotubes by a natural lung surfactant for pulmonary in vitro and in vivo toxicity studies. Part Fibre Toxicol. 2010;19(7):31. 10.1186/1743-8977-7-31 PMC2970581 20958985

[30] Bicer EM . Compositional Characterization of Human Respiratory Tract Lining Fluids for the Design of Disease Specific Simulants. London: King’s College London; 2015.

[31] Kumar A , Terakosolphan W , Hassoun M , et al. A biocompatible synthetic lung fluid based on human respiratory tract lining fluid composition. Pharm Res. 2017;34(12):2454‐2465. 28560698 10.1007/s11095-017-2169-4 PMC5736781

[32] Blachere FM , Lindsley WG , Davis KA , et al. Detection of seasonal and novel H1N1 influenza virus in cough‐generated bioaerosols. Abstracts of the 2010 American Society for Microbiology 110th General Meeting, May 23–27, 2010, San Diego, California. Washington, DC: American Society for Microbiology; 2010 May; Q‐898.

[33] Kalatoor S , Grinshpun SA , Willeke K , Baron P . New aerosol sampler with low wind sensitivity and good filter collection uniformity. Atmos Environ. 1995;29:1105‐1112.

[34] Rauprich P , Möller O , Walter G , Herting E , Robertson B . Influence of modified natural or synthetic surfactant preparations on growth of bacteria causing infections in the neonatal period. Clin Diagn Lab Immunol. 2000;7(5):817‐822. 10973461 10.1128/cdli.7.5.817-822.2000 PMC95962

[35] Slade R , Crissman K , Norwood J , Hatch G . Comparison of antioxidant substances in bronchoalveolar lavage cells and fluid from humans, guinea pigs, and rats. Exp Lung Res. 1993;19(4):469‐484. 8370346 10.3109/01902149309064358

[36] Sun G , Crissman K , Norwood J , Richards J , Slade R , Hatch GE . Oxidative interactions of synthetic lung epithelial lining fluid with metal‐containing particulate matter. Am J Physiol Lung Cell Mol Physiol. 2001;281(4):L807‐L815. 11557584 10.1152/ajplung.2001.281.4.L807

[37] Robinson MD , McCarthy DJ , Smyth GK . edgeR: a Bioconductor package for differential expression analysis of digital gene expression data. Bioinformatics. 2010;26(1):139–140. 19910308 10.1093/bioinformatics/btp616 PMC2796818

[38] Leppänen H , Täubel M , Jayaprakash B , Vepsäläinen A , Pasanen P , Hyvärinen A . Quantitative assessment of microbes from samples of indoor air and dust. J Expo Sci Environ Epidemiol. 2018;28:231‐241. 28975927 10.1038/jes.2017.24

[39] Haugland R , Varma M , Wymer L , Vesper S . Quantitative PCR analysis of selected Aspergillus, Penicillium and Paecilomyces species. Syst Appl Microbiol. 2004;27:198‐210. 15046309 10.1078/072320204322881826

[40] Haugland RA , Siefring SC , Wymer LJ , Brenner KP , Dufour AP . Comparison of Enterococcus measurements in freshwater at two recreational beaches by quantitative polymerase chain reaction and membrane filter culture analysis. Water Res. 2005;39:559‐568. 15707628 10.1016/j.watres.2004.11.011

[41] Kärkkäinen PM , Valkonen M , Hyvärinen A , Nevalainen A , Rintala H . Determination of bacterial load in house dust using qPCR, chemical markers and culture. J Environ Monit. 2010;12:759‐768. 20445866 10.1039/b917937b

[42] Haugland RA , Vesper SJ . Identification and Quantification of Specific Fungi and Bacteria: US Patent 6 387 652. Washington, DC: US Patent and Trademark Office; 2002.

[43] Caporaso JG , Lauber CL , Walters WA , et al. Global patterns of 16S rRNA diversity at a depth of millions of sequences per sample. Proc Natl Acad Sci USA. 2011;108:4516‐4522. 20534432 10.1073/pnas.1000080107 PMC3063599

[44] Jayaprakash B , Adams RI , Kirjavainen P , et al. Indoor microbiota in severely moisture damaged homes and the impact of interventions. Microbiome. 2017;5(1):138. 29029638 10.1186/s40168-017-0356-5 PMC5640920

[45] Callahan BJ , McMurdie PJ , Rosen MJ , Han AW , Johnson AJA , Holmes SP . DADA2: high‐resolution sample inference from Illumina amplicon data. Nat Methods. 2016;13(7):581‐583. 27214047 10.1038/nmeth.3869 PMC4927377

[46] Quast C , Pruesse E , Yilmaz P , et al. The SILVA ribosomal RNA gene database project: improved data processing and web‐based tools. Nucleic Acids Res. 2013;41(D1):D590‐D596. 23193283 10.1093/nar/gks1219 PMC3531112

[47] Caporaso JG , Kuczynski J , Stombaugh J , et al. QIIME allows analysis of high‐throughput community sequencing data. Nat Methods. 2010;7(5):335‐336. 20383131 10.1038/nmeth.f.303 PMC3156573

[48] Mercer EH . A scheme for section staining in electron microscopy. J Royal Microscopical Soc. 1963;81(3‐4):179–186.

[49] Burns WA .Thick Sections: Technique and Applications, Diagnostic Electron Microscopy, Ch. 4, Trump BF, Jones RJ, eds. New York: John Wiley & Sons; 1978.

[50] Shapiro SS , Wilk MB . An analysis of variance test for normality (complete samples). Biometrika. 1965;52(3–4):591‐611.

[51] Dunn OJ . Estimation of the means for dependent variables. Ann Mathemat Statist. 1958;29(4):1095‐1111.

[52] Qiagen . https://www.qiagen.com/us/shop/sample-technologies/rna/qiaseq-targeted-rna-panels/?catno=RHS-006Z#geneglobe. Accessed May 10, 2019.

[53] UniProt Consortium . https://www.uniprot.org/uniprot/P20702. Accessed January 5, 2019.

[54] UniProt Consortium . https://www.uniprot.org/uniprot/B2R4C5. Accessed January 5, 2019.

[55] Pradhan D , Morrow J . The spectrin‐ankyrin skeleton controls CD45 surface display and interleukin‐2 production. Immunity. 2002;17(3):303‐315. 12354383 10.1016/s1074-7613(02)00396-5

[56] Norment AM , Lonberg N , Lacy E , Littman DR . Alternatively spliced mRNA encodes a secreted form of human CD8 alpha. Characterization of the human CD8 alpha gene. J Immunol. 1989;142(9):3312‐3319. 2496167

[57] Gaudet P , Livstone MS , Lewis SE , Thomas PD . Phylogenetic‐based propagation of functional annotations within the Gene Ontology consortium. Brief Bioinform. 2011;12(5):449‐462. 21873635 10.1093/bib/bbr042 PMC3178059

[58] Allam A , Illges H . Calyculin A inhibits expression of CD8alpha but not CD4 in human peripheral blood T cells. Immunobiology. 2000;202(4):353‐362. 11131152 10.1016/s0171-2985(00)80039-x

[59] UniProt Consortium . https://www.uniprot.org/uniprot/P04798. Accessed January 5, 2019.

[60] Moorthy B , Chu C , Carlin DJ . Polycyclic aromatic hydrocarbons: from metabolism to lung cancer. Toxicol Sci. 2015;145(1):5‐15. 25911656 10.1093/toxsci/kfv040 PMC4408964

[61] Campalans A , Amouroux R , Bravard A , Epe B , Radicella JP . UVA irradiation induces relocalisation of the DNA repair protein hOGG1 to nuclear speckles. J Cell Sci. 2007;120:23‐32. 17148573 10.1242/jcs.03312

[62] UniProt Consortium . https://www.uniprot.org/uniprot/O15527. Accessed January 5, 2019.

[63] de Souza‐Pinto NC , Maynard S , Hashiguchi K , Hu J , Muftuoglu M , Bohr VA . The recombination protein RAD52 cooperates with the excision repair protein OGG1 for the repair of oxidative lesions in mammalian cells. Mol Cell Biol. 2009;29(16):4441‐4454. 19506022 10.1128/MCB.00265-09 PMC2725742

[64] Rhodin JAG . The ciliated cell: ultrastructure and function of the human tracheal mucosa. Am Rev Respir Dis. 1966;93(3):1‐15. 5954680 10.1164/arrd.1966.93.3P2.1

[65] Karp G . Cell and Molecular Biology: Concepts and Experiments. New York: John Wiley Sons, Inc.; 2005.

[66] Hill EM , Ling T , Nettesheim P . Differentiation dependency of eicosanoid enzyme expression in human tracheobronchial epithelial cells. Toxicol Lett. 1998;96(97):239‐244. 9820673 10.1016/s0378-4274(98)00078-2

[67] Oshima T , Gedda K , Koseki J , et al. Establishment of esophageal‐like non‐keratinized stratified epithelium using normal human bronchial epithelial cells. Am J Physiol Cell Physiol. 2011;300(6):C1422‐C1429. 21307344 10.1152/ajpcell.00376.2010

[68] Hayden PJ , Klausner M , Kubilus J , Jackson R , Roemer EJ . Reproducibility of Epiairway™, A Differentiated Airway Tissue Model for Pre‐clinical Drug Development Studies. Ashland: MatTek Corporation; 2018.

[69] Dvorak A , Tilley AE , Shaykhiev R , Wang R , Crystal RG . Do airway epithelium air‐liquid cultures represent the *in vivo* airway epithelium transcriptome? Am J Respir Cell Mol Biol. 2011;44(4):465‐473. 20525805 10.1165/rcmb.2009-0453OC PMC3095919

[70] Ross AJ , Dailey LA , Brighton LE , Devlin RB . Transcriptional profiling of mucociliary differentiation in human airway epithelial cells. Am J Respir Cell Mol Biol. 2007;37(2):169‐185. 17413031 10.1165/rcmb.2006-0466OC

[71] Martinez‐Anton A , Sokolowska M , Kern S , et al. Changes in microRNA and mRNA expression with differentiation of human bronchial epithelial cells. Am J Respir Cell Mol Biol. 2013;49(3):384‐395. 23590309 10.1165/rcmb.2012-0368OC PMC3824051

[72] Qin Q , Wu Q , Wang Y , et al. Effects of cellular differentiation in human primary bronchial epithelial cells: metabolism of 4‐(methylnitrosamine)‐1‐(3‐pyridyl)‐1‐butanone. Toxicol In Vitro. 2019;55:185‐194. 30552994 10.1016/j.tiv.2018.12.006 PMC7953429

[73] Fields WR , Leonard RM , Odom PS , Nordskog BK , Ogden MW , Doolittle DJ . Gene expression in normal human bronchial epithelial (NHBE) cells following *in vitro* exposure to cigarette smoke condensate. Toxicol Sci. 2005;86(1):84‐91. 15858226 10.1093/toxsci/kfi179

[74] Fields WR , Desiderio JG , Putnam KP , Bombick DW , Doolittle DJ . Quantification of changes in c‐myc mRNA levels in normal human bronchial epithelial (NHBE) and lung adenocarcinoma (A549) cells following chemical treatment. Toxicol Sci. 2001;63(1):107‐114. 11509750 10.1093/toxsci/63.1.107

[75] Nevalainen A , Täubel M , Hyvärinen A . Indoor fungi: companions and contaminants. Indoor Air. 2015;25(2):125‐156. 25601374 10.1111/ina.12182

[76] Fromme H . Particulate matter in indoor environments ‐ exposure situation in residences, schools, pubs, and related recreational spaces. Gesundheitswesen. 2006;68(11):714‐723. 17199207 10.1055/s-2006-927248

[77] Täubel M , Rintala H , Pitkäranta M , et al. The occupant as a source of house dust bacteria. J Allergy Clin Immunol. 2009;124(4):834‐840. 19767077 10.1016/j.jaci.2009.07.045

[78] Ruzer LS , Harley NH . Aerosols handbook: measurement, dosimetry, and health effects. CRC Press. 2013;318‐321.

[79] Rönkkö TJ , Jalava PI , Happo MS , et al. Emissions and atmospheric processes influence the chemical composition and toxicological properties of urban air particulate matter in Nanjing. China. Science of The Total Environment. 2018;639:1290‐1310. 29929296 10.1016/j.scitotenv.2018.05.260

