# Supplementary figures and images for: Human airway construct model is suitable for studying transcriptome changes associated with indoor air particulate matter toxicity
Source: Indoor Air. 2020 Jan 23;30(3):433–44. doi: 10.1111/ina.12637 (PMC7217003; doi:10.1111/ina.12637)

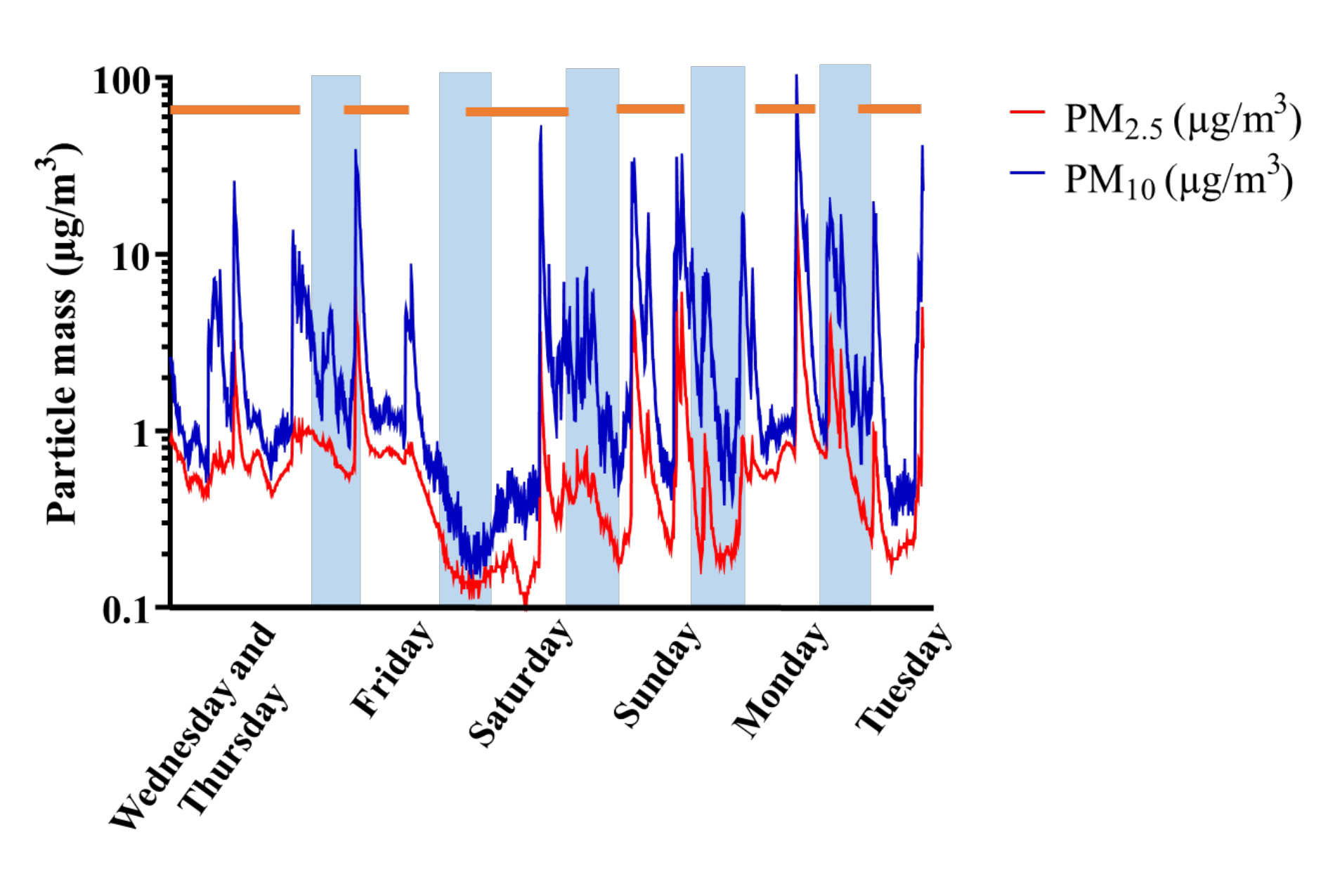

Supplement: Supplementary file 1 [file INA-30-433-s001.jpg]

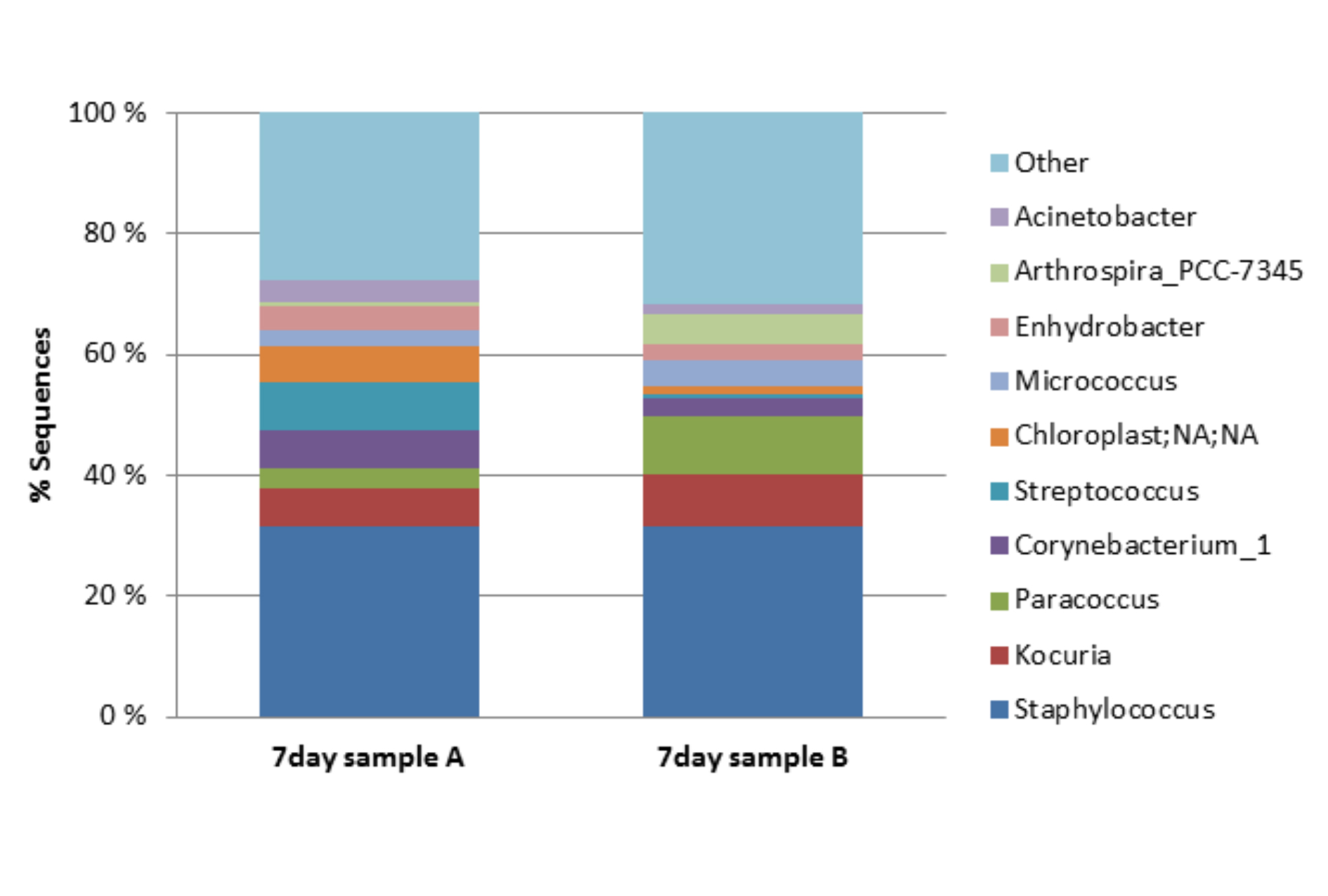

Supplement: Supplementary file 2 [file INA-30-433-s002.jpg]
