# Supplementary material for: Human airway construct model is suitable for studying transcriptome changes associated with indoor air particulate matter toxicity
Source: Indoor Air. 2020 Jan 23;30(3):433–44. doi: 10.1111/ina.12637 (PMC7217003; doi:10.1111/ina.12637)
Supplement: Supplementary file 3 [file INA-30-433-s003.docx]

**Supplementary information**

**Particle concentrations during sampling periods**

The airborne PM concentration varied throughout the day, showing different diurnal cycles for weekend and working days. Particle mass in indoor air decreased during the night and daily work hours, when the occupant was not at home, while we observed increases in the particle concentrations during the active hours of the morning and evening (Figure S1).

Variation in PM_2.5_ and PM_10_ particle mass concentrations (estimated mass concentration based on continuous particle count and mass approximation in the Lighthouse IAQ3016 device) during sampling periods of seven subsequent days are presented in Table S1. Due to the large variation in particle concentrations between individual days, closely linking to occupancy and activity patters in the home, one integrated sample combining sampling periods of seven subsequent days was used for the experiments presented in the main manuscript.

**Microbial content of individual and the integrated sample**

Bacterial and fungal levels of individual 12-hour samples that were collected on seven subsequent days varied considerably between days and were generally very low (Table S1). The bacterial composition as determined via 16S amplicon sequencing varied majorly between days and also in duplicate samples collected within one day, due to low biomass retrieved from the individual Button samples (*data not shown*).

*Table S1. PM_2.5_ and PM_10_ concentrations, microbial levels, and bacterial richness (number of observed sequence variants) in individual daily and the integrated seven-day sampling period.*

| Sample | PM_2.5_ [Mean µg/m^3^] | PM_10_ [Mean µg/m^3^] | Total Bacteria [CE/m^3^] | Total Fungi [CE/m^3^] | Bacterial Richness  [n ASVs] |
| --- | --- | --- | --- | --- | --- |
| Day 1 | 0.63 | 1.93 | 500 | <DL | 17 |
| Day 2 | 0.68 | 2.17 | 394 | 28 | 26 |
| Day 3 | 1.03 | 3.79 | 216 | 9 | 18 |
| Day 4 | 0.32 | 2.34 | 12 | 15 | 14 |
| Day 5 | 1.05 | 6.38 | 274 | 66 | 13 |
| Day 6 | 1.63 | 6.47 | 512 | 42 | 11 |
| Day 7 | 0.34 | 2.36 | 768 | 23 | 9 |
| Seven-day Integrate | 0.81 | 3.63 | 474 | 55 | 76 |

The bacterial composition of two parallel seven-day integrated samples is shown in Figure S2. Samples were dominated by *Staphylococcus* and several other genera likely of human origin (skin and oral sources).

*Figure S1. PM_2.5_ and PM_10_ estimated particle mass (µg/m^3^) during seven days sampling period. Night hours (22.00-7.00) are indicated with light blue background and sampling periods with orange lines. Night-hour data for Wed-Thu night is not included due to technical problems in counting.*

*Figure S2. Top ten most abundant genera in two air samples (A and B) collected in parallel over a period of seven days in the study residence. “Other” refers to the sum of relative abundances of bacterial taxa other than the top ten most abundant genera.*
